# Secondary Metabolites from Gorgonian Corals of the Genus *Eunicella*: Structural Characterizations, Biological Activities, and Synthetic Approaches

**DOI:** 10.3390/molecules25010129

**Published:** 2019-12-28

**Authors:** Dario Matulja, Maria Kolympadi Markovic, Gabriela Ambrožić, Sylvain Laclef, Sandra Kraljević Pavelić, Dean Marković

**Affiliations:** 1 Department of Biotechnology, University of Rijeka, Radmile Matejčić 2, 51000 Rijeka, Croatia; dario.matulja@biotech.uniri.hr; 2 Department of Physics and Centre for Micro- and Nanosciences and Technologies, University of Rijeka, Radmile Matejčić 2, 51000 Rijeka, Croatia; maria.kolympadi@uniri.hr (M.K.M.); gabriela.ambrozic@uniri.hr (G.A.); 3 Laboratoire de Glycochimie, des Antimicrobiens et des Agroressources (LG2A) UMR CNRS 7378—Institut de Chimie de Picardie FR 3085, Université de Picardie Jules Verne, 33 rue Saint Leu, FR-80039 Amiens CEDEX, France; sylvain.laclef@u-picardie.fr

**Keywords:** *Eunicella* genus, eunicellin-type diterpenes, sterols, nitrogenated compounds, cytotoxic, anti-inflammatory, antimicrobial, synthetic approaches

## Abstract

Gorgonian corals, which belong to the genus *Eunicella*, are known as natural sources of diverse compounds with unique structural characteristics and interesting bioactivities both in vitro and in vivo. This review is focused primarily on the secondary metabolites isolated from various *Eunicella* species. The chemical structures of 64 compounds were divided into three main groups and comprehensively presented: a) terpenoids, b) sterols, and c) alkaloids and nucleosides. The observed biological activities of depicted metabolites with an impact on cytotoxic, anti-inflammatory, and antimicrobial activities were reviewed. The most promising biological activities of certain metabolites point to potential candidates for further development in pharmaceutical, cosmetic, and other industries, and are highlighted. Total synthesis or the synthetic approaches towards the desired skeletons or natural products are also summarized.

## 1. Introduction

Oceans and seas cover more than 70% of the surface of our planet and, therefore, are an extensive and rich source of biotechnological potential [1]. Terrestrial organisms, mainly plants, have been widely explored and utilized to obtain novel compounds with the purpose to develop novel drugs for improved treatment for various diseases [2]. In contrast, species living in the marine environment have traditionally been used as food, contributing to human health as valuable sources of proteins, polysaccharides, amino acids, vitamins, minerals, and other micronutrients [1,3]. More than 30,000 compounds have been isolated from marine organisms since biochemical research began in the second half of the last century [4,5]. Numerous sea bioprospecting programs have been performed with the aim to search for new compounds as target leads in the drug discovery processes, emphasizing on anticancer products with improved performance and/or less severe adverse effects [2,6]. Consequently, since 1969 and the approval of cytarabine, the first marine-derived product for the treatment of leukemia, six more compounds have been approved by the Food and Drug Administration (FDA) for clinical use, while dozens of others are currently in different stages of preclinical and clinical trials [7]. Besides anticancer activity, marine natural compounds have also demonstrated neuroprotective, anticardiovascular, antimicrobial, anti-inflammatory, antioxidant, antifouling, and other interesting biological activities [1].

Marine sponges from the phylum Porifera are recognized among the most interesting organisms in bioprospecting as sources of the largest number of so far characterized marine metabolites. Other invertebrates like cnidarians include more than 11,000 species, some of which are poorly or completely unexplored [4]. Moreover, corals are interesting sedentary organisms that may survive harsh internal and external marine environmental conditions. The main reason why they produce various secondary metabolites is to protect themselves from predatory species [4,8]. These characteristics may be exploited in search of novel bioactives with still unexplored biological mechanisms of action.

**Figure 1:**
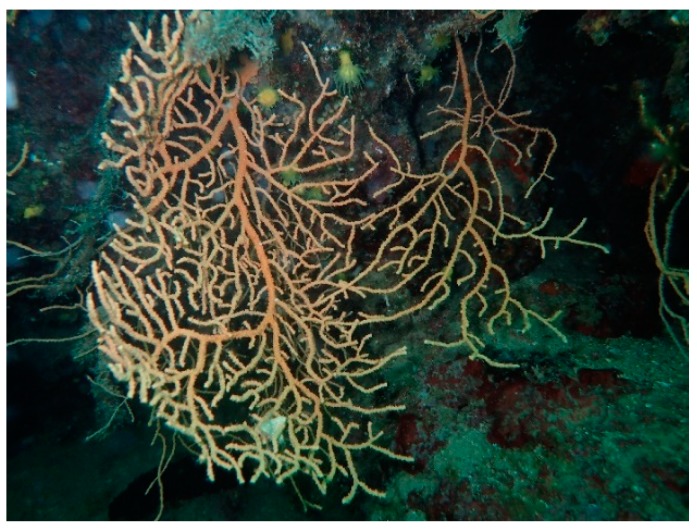
*Eunicella cavolini* from the Adriatic Sea (Rtina, Paška vrata, Croatia), geographical coordinates: Geographical longitude 44°19′14′’, geographical latitude 15°55′42′’.

*Eunicella* coral species belong to the order Alcyonacea, family Gorgoniidae. Gorgonian corals, also known as sea fans and soft corals, are characterized by their branched, fan-like structure which can be found on both hard and soft sea bottoms in the Mediterranean, tropical, and subtropical marine areas [8,9]. Due to their tree-like morphological features, they provide nutrients and protection to other marine invertebrates and vertebrates [10]. For example, bacteria of the genus *Endozoicomonas* are key endosymbionts within colonies of *Eunicella cavolini* (Figure 1) and may contribute to the coral’s adaptation to the environment or to its metabolism [9,11].

Recently, Raimundo et al. have analyzed genomes of 15 bacteria from 12 genera, cultivated from *Eunicella* sp., and identified gene clusters responsible for the biosynthesis of terpenes, polyketides, and peptides [12]. The microorganisms might be also involved in symbiotic biosyntheses producing the different oxygenation and unsaturation patterns of the natural products originating from *Eunicella* species. Furthermore, those symbiotic organisms can be analyzed by a metagenomic approach, which allows the bioprospecting of genes encoding enzymes or discovering of biocatalysts for the synthesis of bioactive secondary metabolites [13,14,15].

*Eunicella cavolini* (yellow gorgonian, Koch, 1887) and *Eunicella singularis* (white gorgonian, Esper, 1791) are the most abundant gorgonian corals in the Mediterranean marine area [10]. The first is recognized by its orange-colored colonies and is widespread from the Tunisian coast to the Aegean Sea. It can be found up to 150 m where it can form larger, more arborescent colonies due to the environmental stability [10,16]. Other *Eunicella* members mentioned in this review are *granulata* (Grasshoff, 1992), *verrucosa* (Pallas, 1766), and *labiata* (Thomson, 1927) [17]. In addition to their potent biological activities, high susceptibility to climate change, anthropogenic influences, and other abiotic and biotic factors, together with their slow growth and recovery rates, make the *Eunicella* sp. A very interesting subject in different research areas.

Two previous reviews cover the isolation of bioactive metabolites from gorgonian species between 2009 and 2012 [8,18]. Herein, we summarize advances in isolation and characterization of all known compounds from *Eunicella* sp. during the last three decades with a focus on the research conducted from 2013 to present. The presented compounds were divided into three groups, i.e., (1) terpenoids, (2) sterols, and (3) alkaloids and nucleosides. Biogenetic pathways involved in the formation of terpenoids from geranylgeranyl diphosphate are also described. In connection to the diversity and complexity of isolated structures, their biological effects are discussed, highlighting cytotoxic, anti-inflammatory, and antimicrobial activities. The most interesting or promising compounds and their biological activities are summarized in Tables 1–3. Moreover, synthetic approaches and total syntheses of isolated metabolites are schematically depicted.

## 2. Terpenoids

### 2.1. Biogenesis and Structural Analysis of Eunicellins

**Figure 2:**
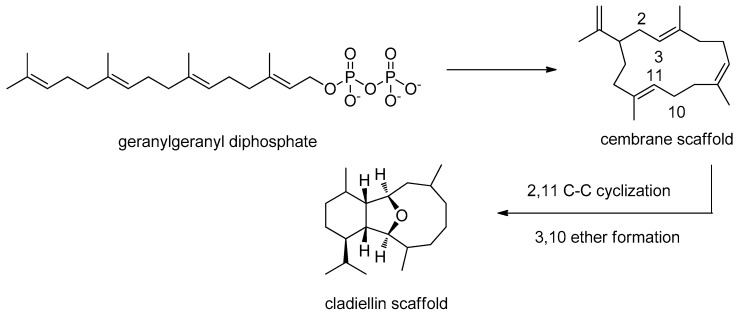
Biosynthetic pathway to cembranoid and cladiellin cores [23].

Among all metabolites isolated from gorgonian soft corals, those belonging to the class of terpenoids exhibited the most significant activities (IC_50_ values lower than 5 μg/mL) when assayed in vitro against tumor cell lines [4]. Diterpenes make up to 65% of all published metabolites from gorgonian natural resources [19]. *Eunicella* sp. are producers of eunicellin-type diterpenoids, i.e., eunicellin-based diterpenoids from the gorgonian *Eunicella verrucosa* [20] or from *Eunicella cavolini* [21] and *Eunicella labiata* [22]. These compounds have the cladiellane skeleton and contain four isoprene units linked head to tail (Figure 2). According to Welford et al., cladiellins are formed by cyclization of geranylgeranyl diphosphate, C2–C11 cyclization, and C3–C10 tetrahydrofuranyl formation from cembrane scaffold intermediate [23].

**Figure 3:**
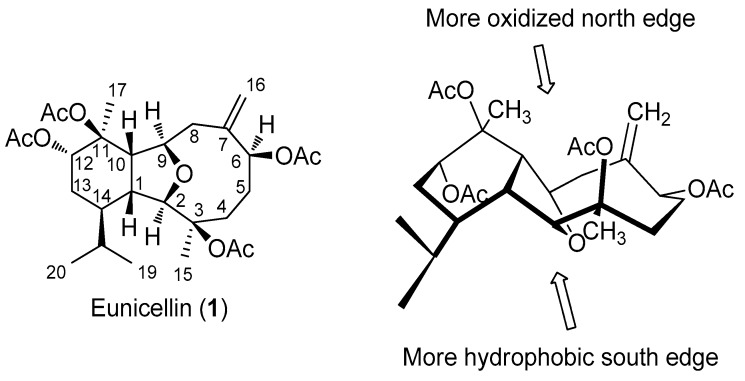
Eunicellin (**1**).

Eunicellin (1), shown in Figure 3, possess a tricyclic 6-5-9 membered structure with etheral bridge. The skeleton can be divided into the more oxidized north edge, and the more hydrophobic south edge that communicate through a tricyclic structure. Whereas the west part of the molecule is more rigid due to a bicyclic cyclohexyl-tetrahydrofuranyl system, the eastern part is more flexible as it contains an oxonane structure. A cyclohexyl ring is fused to a tetrahydrofuranyl ring through C1 and C10 pointing hydrogens syn to each other and anti to the H-atoms of the ethereal O-C2 and O-C9 bonds. Examples of deviations of this common oxygen bridge can also be found, from no ether bridge, 2,6-epoxy bridge, to compounds having two epoxy bridges. Angular methyl groups at C3 and C11 are anti-periplanar to the isopropyl group at C14, whereas at C7 is placed methylene group. Eunicellin (1) possess nine sterocenters. C1, C2, C9, and C10 form the central bridging tetrahydrofuranyl system, and still C3, C6, C11, C12, and C14 are chiral. Four acetoxy groups are placed on C3, C6, C11, and C12. Eunicellins also bear different oxygenation and unsaturation patterns with one or more common acetoxyl moieties [22,24,25,26,27,28].

### 2.2. Structural Diversity of Terpenoids from Eunicella sp.

**Figure 4:**
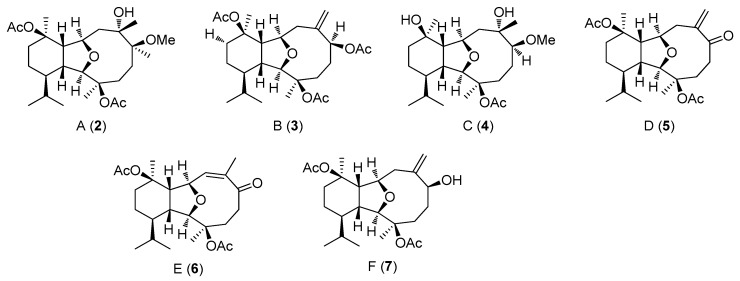
Palmonines A–F (**2**–**7**) isolated by Ortega’s group.

More than 40 eunicellan-type diterpenes have been isolated from gorgonians so far. However, few studies have also included evaluation of the biological activity of these compounds [8,19]. The first such metabolite was isolated in 1968 by Kennard et al., named eunicellin (**1**), having four acetyl groups attached to cladiellane skeleton (Figure 3) [25]. In 1993, Ortega et al. reported the isolation of five new diterpenoids **2**–**6** from the methanolic extract of *Eunicella verrucosa* collected near the Spanish coastal village of Palmones and named palmonines A–E (**2**–**6**), after the sampling site of gorgonian (Figure 4). The latter diterpenoid 6 formed white crystals, while the rest of the compounds were isolated as colorless oils. Apart from the acetate groups, they also differ in some other structural features. For example, palmonine D (**5**) and E (**6**) are two isomers bearing a conjugated keto group with an exocyclic or endocyclic double bond, respectively [20]. The same authors isolated another eunicellin-type diterpenoid from the same octocoral, palmonine F (**7**), which is a derivative of palmonine B (**3**) but displaying one less of acetoxyl NMR signals. They also performed oxidation of the secondary hydroxyl group of palmonine F (**7**) by Jones reagent, which led to the semisynthesis of palmonine D (**5**).

**Figure 5:**
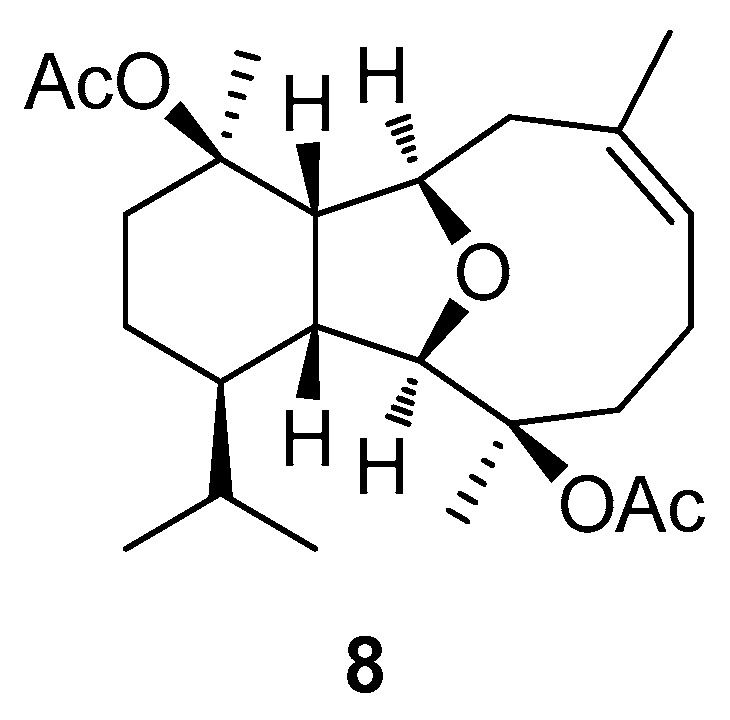
Diterpenoid (**8**) isolated by Rosa et al. from *E. cavolini*.

From 2013 to 2017, Deghrigue’s group and Ioannou’s group conducted thorough studies on *E. singularis* and *Eunicella cavolini* comprising preliminary biological evaluation of the organic extracts and fractions. It was found that the polar fractions show anti-inflammatory and analgesic potentials, as well as anti-proliferative effects on breast cancer cells MCF-7 and prostate cancer cells LNCaP, indicating the presence of novel bioactive compounds [19,29,30,31,32]. The organic extracts of the coral were prepared by maceration and were examined for the presence of several class of compounds, i.e., polyphenols, alkaloids, saponins, steroids, terpenoids, and glycosides by qualitative chemical screening tests based on visible chemical change [29,31]. Final purification of fractions resulted in isolation of 2 terpenoids, palmonines D (**5**) and F (**7**), and nine steroids of various chemical structures (Section 2.3., vide infra) [30,32]. Moreover, Rosa et al. first isolated diterpenoid **8** bearing two acetoxyl groups, from the *E. cavolini* acetone extract (Figure 5) [21].

**Figure 6:**
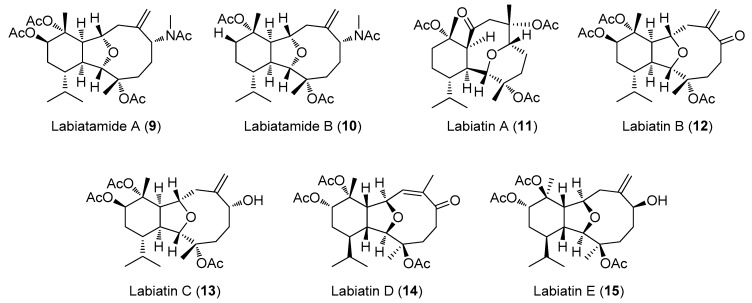
Labiatamides A (**9**) and B (**10**) and labiatins A–E (**11**–**15**) isolated from *E. labiata*.

Another Senegalese, deep-water gorgonian coral, *E. labiate,* was used to extract five diterpenoids with unique structural characteristics from the brown organic extract obtained by maceration of the natural material with dichloromethane and methanol (9:1). Roussis et al. reported the structures of labiatamides A (**9**) and B (**10**), as well as labiatins A–C (**11**–**13**) (Figure 6). These compounds have an isopropyl group and a large number of acetoxyl groups, a characteristic which is also found in palmonines, mentioned previously. Labiatamides A (**9**) and B (**10**) possess a *N*-methyl acetamide moiety, while labiatin A (**11**) contains an unusual ether bridge between C2 and C6 [26]. Furthermore, Kakonikos et al. also isolated two new labiatins D (**14**) and E (**15**) from *E. labiata* organic extract (Figure 6). An eunicellin-type diterpenoid, labiatin D (**14**) is a colorless oil and showed similar spectroscopic characteristics as palmonine E (**6**) with one more acetyl carbonyl moiety. Labiatin E (**15**), a yellowish oil, also has 3 acetyl groups and spectroscopically is identical to previously isolated labiatin C (**13**), with the only difference being the stereochemistry of hydroxyl group at C6 position [22].

**Figure 7:**
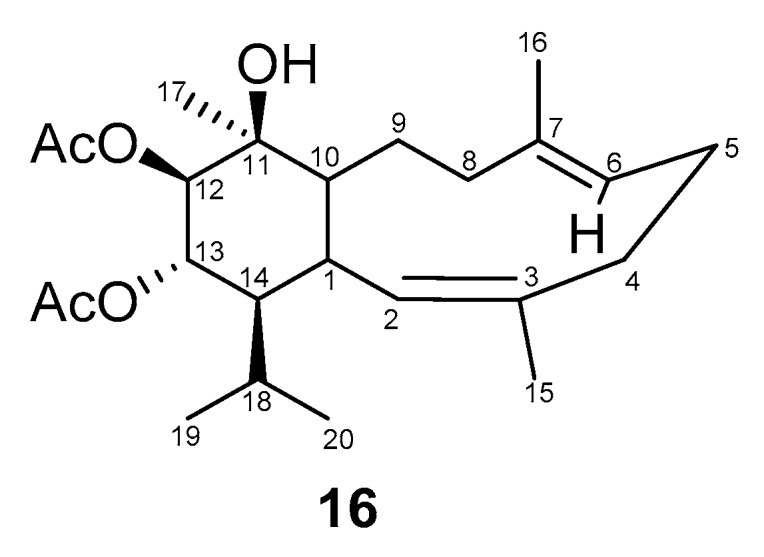
The 12,13-diacetoxycladiella-2,6-dien-11-ol (**16**) isolated from *E. labiata*.

So far, all mentioned diterpenoids contain the cladiellane carbon skeleton (vide supra). However, there is an example of a metabolite having the same structure without an ethereal O-C2 and O-C9 bridge. The 12,13-Diacetoxycladiella-2,6-dien-11-ol (**16**) was isolated from *E. labiata* by Ortega et al. in 1997 (Figure 7). This secondary metabolite 16 possesses two acetoxyl groups and one hydroxyl group. The endocyclic double bond at C2 position has cis stereochemistry, whereas the one at C6 position is trans [24].

**Figure 8:**
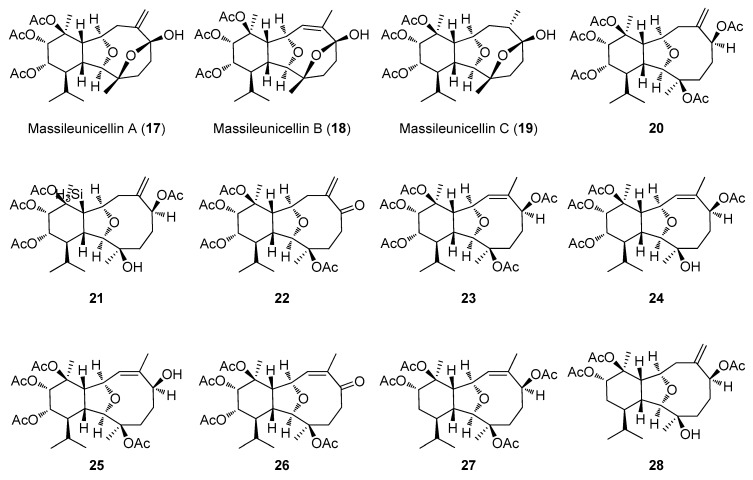
Massileunicellins A–C (**17**–**19**) and diterpenoids (**20**–**26**) isolated from *E. cavolini*, as well as (**27**, **28**) isolated from *E. singularis*.

Two gorgonians, *E. cavolini* and *E. singularis*, were collected near Marseille and extracted with ethanol by Mancini et al., leading to the isolation of, in total, 20 compounds (Figure 8). Some of them, like palmonines D (**5**) and E (**6**), labiatins B–D (**12**–**14**) and eunicellin (**1**) were already characterized by other scientific groups and discussed above (vide supra). Additionally, various oxidation numbers at the 10-membered ring and a second epoxy bridge or a differently located double bond can be observed in massileunicellins A–C (**17**–**19**) [27,28]. The isolation, conformations, and reactivities of diterpenoids **20**–**26** isolated from *E. cavolini*, and diterpenoids **27** and **28** from *E. singularis* are discussed by Mancini et al. emphasizing the various oxygenation patterns of cyclohexane ring [28].

### 2.3. Steroids

There have been several reports on isolation, characterization, and biological evaluation of various steroids from members of the *Eunicella* genus with unique structural features. These molecules differ in their degrees of oxygenation, as well as side chain patterns, which could be the reason for their miscellaneous activity, both in vitro and in vivo [19,30,31,32,33,34,35].

**Figure 9:**
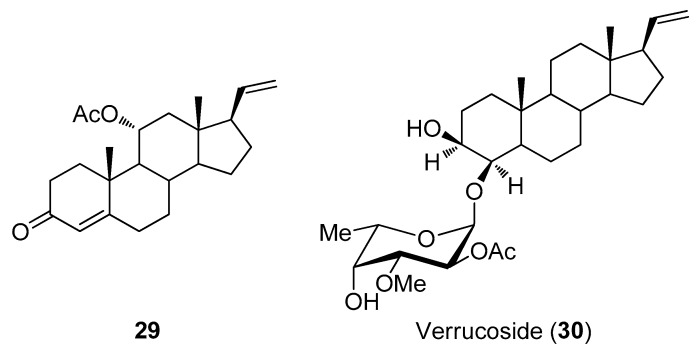
Pregna-4,20-dien-11α-ol-3-one acetate (**29**) and verrucoside (**30**).

The acetone extract of *E. cavolini* was used to isolate a novel steroid, pregna-4,20-dien-11α-ol-3-one acetate (**29**), a pregnane derivative containing vinyl group. Besides spectroscopic elucidation, the authors also presented a chemical synthesis of **29** starting from a progesterone derivative (Figure 9) [35]. It should also be mentioned that Kashman et al. extracted verrucoside (**30**), a pregnane glycoside, after studying the cytotoxic activity of the DCM/MeOH extract of another gorgonian, *E. verrucosa*. This compound is known for having a 6-deoxy hexose derivative as a sugar unit, and Δ^20^ pregnane skeleton as aglycone [36].

**Figure 10:**
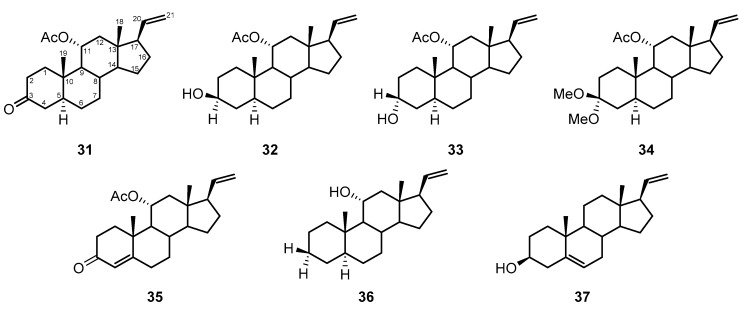
Structures of pregnanes (**31**–**37**).

More pregnanes were extracted from *E. cavolini* within a comprehensive study by Ioannou et al. to identify new natural compounds from Greek marine organisms in 2008 (Figure 10). Seven pregnanes **31**–**37** were reported whose absolute stereochemistries were determined by Mosher’s method. Their structures differ in moieties at C3 and C11 positions of pregn-20-ene skeleton having acetoxy, methoxy, or hydroxy groups. The authors also pointed to the possibility of **34** being the product of reaction with methanol, a solvent which was used for both extraction and purification [37].

**Figure 11:**
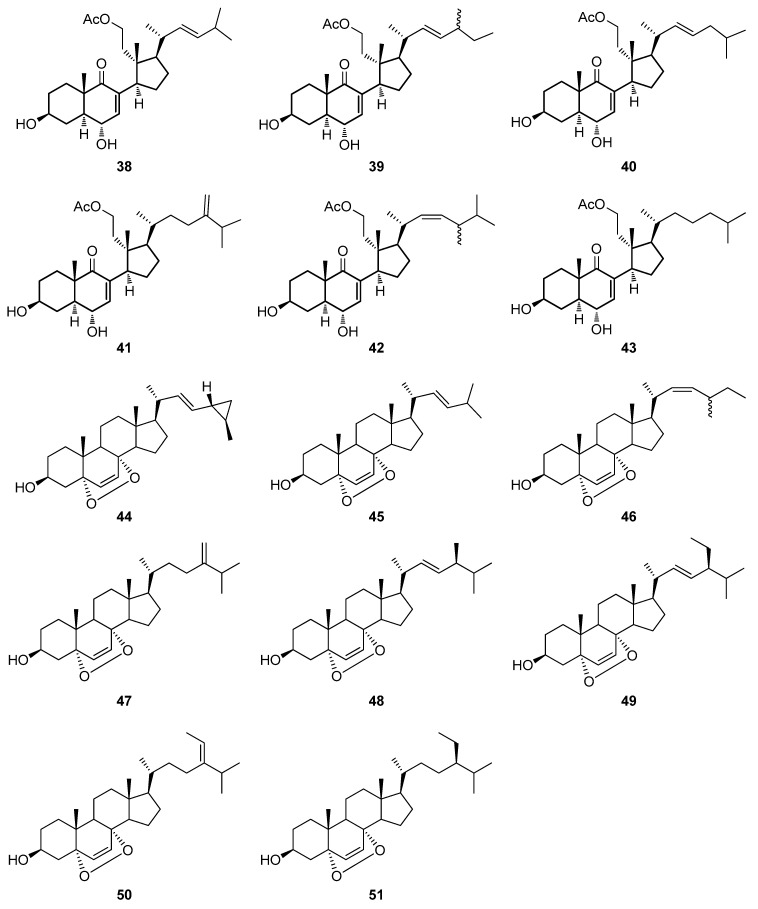
Structures of nine 11-secosterols (**38**–**43**) and 5α,8α-epidioxysterols (**44**–**51**).

The same authors went on the isolation of steroids with more complex structures. Six 9,11-secosterols **38**–**43** and eight 5α,8α-epidioxysterols **44**–**51** were reported from the same gorgonian species conserving the steroidal carbon skeleton but having different aliphatic side chains (Figure 11). The first group of compounds showed C9–C11 bond cleavage in tetracyclic nucleus, while the latter one had higher oxygenation levels due to the presence of an endoperoxide bond. Those characteristic features are often found in marine invertebrates, particularly soft corals [33,34,38,39]. The 5α,8α-endoperoxides **44**–**51** are oxidized sterols which can be formed during photooxidation reaction by addition of an oxygen molecule to a conjugated 5,7-diene system in the precursor molecule which contains the steroidal core [40,41,42,43]. This 5α,8α-endoperoxide bond is exactly responsible for the observed biological activities [40]. Sterol 48 has a structure almost identical to ergosterol peroxide, one of the most characteristic epidioxysteroidal derivatives found in both marine and terrestrial organisms. The only difference between **39** and ergosterol peroxide is the C24 stereogenic center of *S*- and *R*-configuration, respectively [42,44,45,46]. Gorgonian *Verrucella umbraculum* from the South China Sea is also a natural source of sterols **48**, **49**, and **52 [47]**. Interestingly, **44** stands out from the rest of epidioxysterols found in *Eunicella* sp., due to the presence of a cyclopropyl moiety at the side chain [33,34].

**Figure 12:**
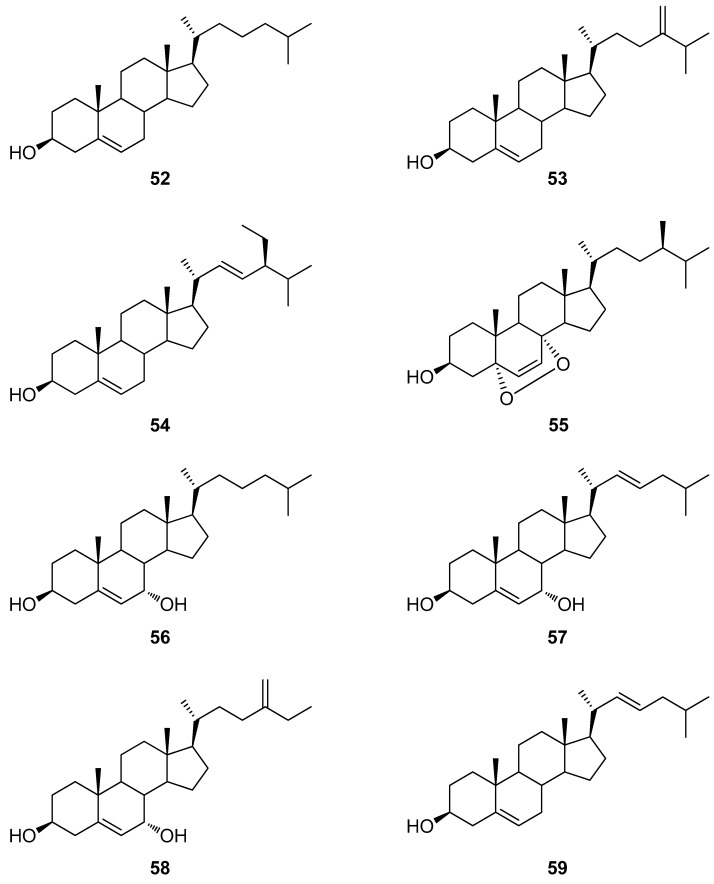
Structures of steroids (**52**–**59**) isolated from the Tunisian gorgonian coral *E. singularis*.

As mentioned previously in Section 2.1. (vide supra), together with various classes of natural products, Deghrigue et al. and Lajili et al. extracted nine mono- or polyhydroxylated steroids from the Tunisian gorgonian coral *E. singularis*, **48** (Figure 11) and **52**–**59** (Figure 12), of which two were epidioxysterols. Besides the number of hydroxyl groups and oxygenation of steroidal core, these products also differ in saturation patterns of side chain which was confirmed by comparison of 1D and 2D NMR data with the literature [19,30,32]. Steroid **56** was also found in other marine species, soft coral *Dendronephthya gigantean* and marine bryozoans, *Biflustra grandicella,* and *Cryptosula pallasiana* [48]. Furthermore, compounds **51** and **55** were also isolated from the tunicate *Didemnum salary* by Bensemhoun et al. [49].

### 2.4. Alkaloids and Nucleosides

About 2% of metabolites isolated from all gorgonian species contain an nitrogen atom including alkaloids, nucleosides, amide, and tryptamine derivatives [8,50,51,52]. As with steroids and terpenoids, nitrogen containing compounds contribute to organisms by acting as a defense against negative external influences, increasing the biodiversity of marine habitat [50,51]. Particularly, xanthines and their methylated derivatives are often found in marine organisms included in nucleotide metabolism [50]. They have already been isolated from other corals, particularly of the genus *Sinularia*, exhibiting antibacterial and cardiotonic activities [53,54]. Samori et al. reported the concentration of N-heterocyclic compounds to be differently obtained following extraction of several gorgonian species with CH_2_Cl_2_/MeOH mixtures or acetonitrile during optimization of the extraction protocol for more polar metabolites. Furthermore, they observed no significant difference in the presence of nitrogen containing metabolites in three *Eunicella* species (*verrucosa*, *cavolini,* and *singularis*). They extracted hypoxanthine, guanine, and adenosine, the latter being absent in *E. verrucosa* and guanine being the most abundant [50].

**Figure 13:**
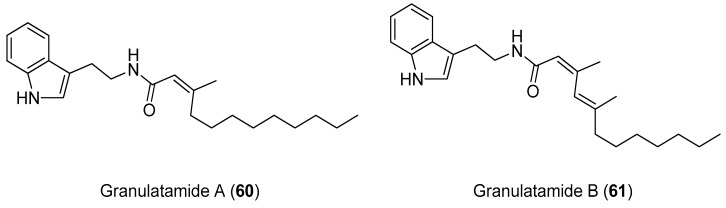
Structures of granulatamides A (**60**) and B (**61**) isolated from *E. granulata* collected in Senegal.

Gorgonian *E. granulata* collected in Senegal was the subject of the study led by Reyes et al. to find new compounds with antitumor activity (Figure 13). The 2-propanol coral extract afforded granulatamides A (**60**) and B (**61**) after semipreparative, reverse-phased HPLC. Both novel compounds contained a tryptamine moiety connected to a fatty acid and differ in the number of double bonds in the aliphatic chain. These compounds exerted only a moderate effect in vitro against different tumor cell lines [51].

**Figure 14:**
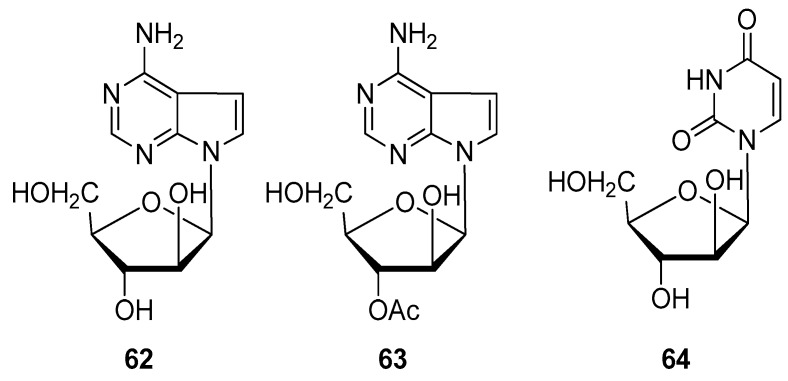
Structures of nucleosides 9-β-d-arabinofuranosyladenine (araA) (**62**), its 3′-*O*-acetyl derivative (**63**), and spongouridine (araU) (**64**) isolated from *Eunicella cavolini*.

The n-butanolic extract of *Eunicella cavolini* was found to be a natural source of the polar nucleosides: 9-β-d-arabinofuranosyladenine (araA) (**62**), its 3′-*O*-acetyl derivative (**63**) and spongouridine (araU) (**64**) (Figure 14). The latter had already been isolated from the sponge *Cryptothetia cripta*. However, low solubility and susceptibility to deamination of the natural compounds led to the synthesis of several acyl derivatives that showed better properties in terms of drug administration [52].

## 3. Biological Activities

### 3.1. Cytotoxic Activity

Cnidarians, and, therefore, members of the genus *Eunicella*, are prominent sources of steroids and terpenoids which demonstrated antitumor activities [4]. All palmonines (**2**–**7**) mentioned in Section 2.1. (vide supra) were screened for their cytotoxic effect against several human or murine cancer cell lines: A549, HT29, P-388, and MEL28. The last two, mice lymphoma and human melanoma lines, respectively, were the most affected by **3**, exhibiting IC_50_ values of 5 µg/mL [55]. Similar results were observed with excavatolides O and Q, diterpenoids with a briarane-type skeleton extracted from the gorgonian *Briareum excavatum* [8]. The authors hypothesized that the activity of those metabolites could arise from synergistic effects and interactions with other compounds. Since palmonine B (**3**) has the highest number of acetoxyl groups, that could also play a role in the observed cytotoxic activity [55]. Furthermore, significant anticancer potential of palmonines F (**7**) and D (**5**) was observed by inducing apoptosis of 92% and 93% of cells, respectively, in a breast cancer cell model (MCF-7 monolayer) at concentration of 200 µg/mL. The authors determined EC_50_ values from the concentration-response curves for **7** and **5,** which were 13 µg/mL and 49 µg/mL, respectively. Lajili et al. also investigated the effect of two previously mentioned palmonines on MCF-7 mammospheres and reported reduction in spheroid size, which was also concentration-dependent and visible 2 days after incubation [19].

Labiatins B, C. and E (**12**, **13**, and **15**) were assayed for their cytotoxic effect against human cancer cell lines [22,26]. Labiatin B (**12**) was tested against colon cancer line (HCT-116) and the authors reported an IC_50_ value of 0.85 µg/mL [26]. The last two compounds showed a cytostatic effect against non-small cell lung cancer line (NSCLC-N6) with half inhibitory concentrations of 35 µg/mL and 7.7 µg/mL for labiatin E (**15**) and C (**13**), respectively, indicating that the stereochemistry influences the biological activity of these secondary metabolites [22].

Massileunicellins A–C (**17**–**19**) were additionally assayed for antitumor and antiviral activity against KB and L1210 doxorubicin-resistant tumor cells, and Dengue virus, respectively. After observing only low cytotoxic effects of massileunicellins, Mancini et al. screened labiatin B (**12**) and palmonine D (**5**) against the same cells which also resulted in poor activity. Comparing those results with the significant cytotoxic activity reported by Ortega et al. [55] and Roussis et al. [26], they hypothesized that diterpenoids might be selective for specific tumor cells. Furthermore, they did not observed any stabilization effect of **5** and **12** on tubulin, a feature characteristic for other types of 2,11-cyclized cembranoids, i.e., sarcodictyns and eleutherobin [27]. The most promising cytotoxic diterpenoids isolated and studied from the genus *Eunicella* are shown in Table 1.

Steroid compounds of natural origin have recently attracted considerable attention among the scientific community due to their interference with molecular pathways of MCF-7 cells. It has been shown that steroid derivatives inhibit estrogen-dependent proliferation and survival of those cells binding to estrogen receptor, thus disrupting the interaction with transmembrane growth factor receptor [33,34,37]. Furthermore, according to Chen and his group, secosteroids and polyhydroxylated steroids could be potential inhibitors of protein-tyrosine phosphatase 1B (PTP-1B), which is involved in metabolism of glucose via hydrolyzation of insulin receptor [39,56]. However, PTP-1B was also found to have a contradictory role in cancer biology. Its deficiency has led to the growth of B lymphoma in mice while it had no role in breast tumorigenesis [57]. Nevertheless, it presents a novel target in both cancer and metabolism treatment.

Ioannou et al. assayed the growth inhibitory potential of sterols isolated from *E. cavolini* (**31**–**39**, **41**, **42**, **44**–**51**) against MCF-7 cells cultured with and without a physiological concentration of 17β-estradiol (1 nM) [33,34,37]. When compared to the effect of positive control (ICI 182,780, Faslodex^®^, a commercially available estrogen receptor down-regulator), compounds **31**–**35** inhibited cell growth for 40 to 49%, while the last two pregnanes (**36**, **37**) were almost inactive. According to the authors, the presence of acetoxyl moiety at C11, observed in metabolites with significant inhibitory effect, could play a role in mediating estrogen-dependent growth of MCF-7. Another possible molecular target is the nuclear pregnane X receptor, a target of several marine-derived compounds involved in drugs and xenobiotics metabolism, oxidative stress, cancer formation, and other physiological processes which yet need to be examined [37,58,59,60]. Secosterols **38** and **39** exhibited IC_50_ values of 7.6 and 7.4 μM against MCF-7 cells, respectively. On the other side, growth inhibition by all compounds was significantly enhanced in MCF-7 cells cultivated in the absence of growth factors and endogenous estrogen. Since **38** was also found to be effective against proliferation of K562 and HeLa cells, it is assumed that secosterols’ antiproliferative activity is selective towards specific tumor cells, as well as the applied growth conditions. Furthermore, since tested compounds differ only in the side chain structure, the better biological activities of **38** and **39** could also be attributed to the double bond at C22 position with E geometry [34]. The gorgonian coral, *Subergorgia suberosa*, collected from the South China Sea, was used to extract several secosterols bearing a hydroxyl group at C11, including **38** and **43**. Zhang et al. reported IC_50_ values of 28.1 and 23.3 μM against HeLa cells for **38** and **43**, respectively, and hypothesized that the presence of a free -OH group at C11 does not contribute to the observed cytotoxic effects of the tested compounds [38]. That is also consistent with the conclusion reported by Kongkathip et al. who have synthesized and evaluated the cytotoxicity of 9,11-secosterol derivatives against KB, HeLa, and MCF-7 cells, as well as non-transformed Vero cells. According to the authors, the key to the observed biological activity is in the presence of a cholesterol-like side chain and a keto group at C9 position [61].

Epidioxysterols **44**–**51** also displayed significant inhibition activity against growth of MCF-7 cells in the growth factor and endogenous steroids depleted medium. At a concentration of 10 µM, **44**, **45**, and **46** displayed almost 60% of the effect of positive control, ICI 182,780 (Faslodex^®^). Considering that **44** was particularly effective against MCF-7 cultured in both cell growth medium conditions, the authors assume that the cyclopropyl group at the side chain of **44** was responsible for the observed effects [33]. Luo et al. also isolated **45** from another marine organism, sponge *Topsentia* sp., and observed its moderate cytotoxic activity against different tumor cell lines of human origin: A549, SK-OV-3, SK-MEL-2, XF498, and HCT15 (IC_50_ values: 30.0, 19.4, 25.9, >30.0, 21.4 μg/mL, respectively) [62]. Another marine sponge, *Monanchora* sp., was used in the study conducted by Mun et al. in 2015 who isolated epidioxysterols **45** and **47**. The authors evaluated the activities of these two products against renal, pancreatic, and colorectal cancer cell lines and suggested that the C24 (28) double bond, which is a feature of **47,** might be responsible for its increased cytotoxic activities. At a concentration of 23 μM, **47** induced 50% inhibition of renal A-498 cells. It should be emphasized that **47** exhibited higher cytotoxicity than the positive controls, temsirolimus and 5-fluorouracil, used in the clinical treatment of the above mentioned cancers [63]. The 48 showed significant antiproliferative activity against A549, H460, and HGC27, displaying IC_50_ values of 10.9, 10.9, and 11.6 μg/mL, respectively, as well as moderate cytotoxicity towards human ovarian cells (A2780) with an IC_50_ value of 16 μg/mL [64,65]. Raslan et al. isolated epidioxysterols **49** and **51** from the marine sponge *Monanchora clathrate* but observed only weak cytotoxicity against MES-SA, MCF-7, and HK-2 cancer cell lines with IC_50_ values higher than 25 μg/mL [66].

Deghrigue et al. and Lajili et al. also investigated the cytotoxic activity of steroids isolated from *E. singularis* after observing promising antiproliferative, antioxidant, and other pharmacological activities of its organic extract and fractions [19,30,32]. Metabolites **52**, **54**, and **59** were evaluated for their anticancer activity against a monolayer and a spheroid model of MCF-7 using AnnexinV- fluorescein isothiocyanate/propidium iodide (FITC/PI) flow cytometry and spheroid size analysis, respectively. Cholesta-5,22-diene-3β-ol (**59**) displayed an EC_50_ value of 30 µg/mL against monolayer cells causing apoptosis in 91% of cancer cells at 200 µg/mL. However, steroids were more effective against the spheric model than diterpenoids (**5** and **7**) isolated from the same organism. The reported growth rates for **52**, **54**, and **59** were 1.17, 1.25, and 1.15, respectively, at a concentration of 200 µg/mL and comparable to the control, taxol at 100 µM [19]. On the contrary, according to Tian et al., **52** was inactive in vitro against HL-60, HepG2, and SGC7901 cell lines [67]. Finally, Pan et al. investigated the proapoptotic activity of the ethyl acetate extract of the hard clam *Meretrix lusoria* and consequently isolated a mixture of epidioxysterols containing **55**. The authors observed 35% and 73% apoptotic HL-60 cells after treatment with 25 and 50 µg/mL of this mixture, respectively [68]. The antitumor activities of sterols are summarized in Table 2.

Granulatamides A (**60**) and B (**61**) were both evaluated for their cytotoxic activities against 16 different human cancer cell lines by the use of sulphorhodamine B colorimetric assay (Table 3), exhibiting low GI_50_ (growth inhibition concentration) values of 1.7 and 3.5 µM against two human prostate cancer cell lines, DU-145 and LN-caP, respectively [51]. Another nitrogen containing compound, nucleoside 63 was assayed for cytotoxic activity against KB cells displaying a low IC_50_ value of 5 µg/mL [52].

### 3.2. Anti-Inflammatory Activity

Discovery of novel compounds that may be used to target inflammatory processes is highly needed since the use of many existing nonsteroidal anti-inflammatory drugs have been linked with various severe adverse effects [30,32]. In this context, Deghrigue et al. have reported that the organic extract and fractions obtained by further purification with ethanol, acetone, and dichloromethane/methanol have exhibited anti-inflammatory activity in a dose-dependent manner regarding the inhibition of edema in rats induced by carrageenan. At a dose of 25 mg/kg of the ethanolic semipurified fraction, the edema was inhibited by 66% after 3 h, which is 10% higher than observed with a reference drug, acetylsalicylaze-lysine (ASL). Final purification of the ethanolic fraction led to isolation of owp palmonines, **5** and **7,** and nine sterols, **48**, **52**–**59**, which could contribute to the investigated biological activity [30,32]. Anti-inflammatory activity of epidioxysterols **48** and **55** was further investigated by other scientific groups [68,69,70]. Just recently, Huynh et al. studied the influence of **48** on expression of pro-inflammatory proteins, iNOS and COX-2, released from macrophages. They observed significant suppression of iNOS by **48** at 10 µM. However, dendronesterone D bearing acetoxyl moiety at C11 showed the highest suppression of inflammation [70]. Inhibition of NO production by lipopolysaccharide (LPS)-stimulated macrophages was used to assess anti-inflammatory activity of 55 in a mixture of epidioxysterols by Pan et al. NO production did not arise from the cytotoxic activity and was inhibited by 38%, 39%, 99%, and 100% at concentrations of 5, 10, 25, and 50 µg/mL, respectively [68]. Immunosuppressive activity of **47** and **48** against T and B lymphocyte cells proliferation was also examined by Yang et al. in 2019. Both epidioxysterols were extracted from a soft coral *Sinularia* sp. Reported IC_50_ values of **47** and **48** were 57.54 and 59.54 µM for T cells, and 47.57 and 19.30 µM for B lymphocytes, respectively. Thus, they showed weaker anti-inflammatory activity than cyclosporin A (IC_50_ = 0.04 and 0.40 µM for T and B cells, respectively), however they were less cytotoxic against murine splenocytes [69].

### 3.3. Antimicrobial Activity

Bioactive secondary metabolites from *Eunicella* sp., as well as compounds from other soft corals, showed interesting antibacterial and antiviral activities [4,52,71,72]. According to Gauvin et al., epidioxysterols are the first natural metabolites to display antiviral activity against lymphoma causer, HTLV-I retrovirus. A mixture of sterols, including **49**, **51**, and **55**, reduced by 50% β-galactosidase activity which is correlated to virus activity at a concentration of 0.3 mg/mL. However, they were inactive against another RNA virus, HIV [71]. Polar nucleosides from *E. cavolini*, 9-β-d-arabinofuranosyladenine (spongoadenosine, araA) (**62**) and spongouridine (araU) (**64**), showed promising antiviral activity against DNA viruses [52]. Pharmacological properties and biosynthesis of araA (**53**) have been already reviewed by Huang et al. [73]. It is worth mentioning that **62** was the first drug to treat herpes encephalitis infections and diseases caused by other herpes viruses in patients with impaired immune system [73]. Synthetic *O*-acyl derivatives of **62** showed also promising antiviral effects but its 3′-*O*-acetyl derivative (**63**) yet needs to be examined in order to complete the structure activity relationship studies [52]. We would also like to mention that Liang et al. observed an antibacterial activity of epidioxysterols 47 with MIC_50_ value of 500 µM against *Staphylococcus aureus* Newman strain. This activity is considered significant because the MIC_50_ value is lower than 1 mM [72].

## 4. Synthetic Approaches

One of the major problems during the preclinical and clinical development of naturally derived drugs is the insufficient quantity of the isolated natural product precursors [2,4,74]. As the consequent market application by the pharmaceutical and cosmetic industries entails the desired metabolite to be available often on the ‘kilogram scale’, it is not surprising that some compounds which demonstrated promising biological activities have never reached the market. In order to obtain sufficient amounts of a natural product or its derivatives, either the extraction of the natural row material should be repeatedly performed or the natural product should be synthetized [75]. The extraction pathway may be problematic in terms of the percentage of the desired secondary metabolite in the row material and/or ecological and ethical principles. For example, in the case of *Eunicella* species, mass mortality events have emerged in the past few years mainly hitting the gorgonian corals in the Mediterranean Sea [76,77,78,79,80]. Furthermore, collecting the same species in various geographical areas or during different time seasons might result in a different chemical composition, thereby reducing or increasing the yield of the desired product [75,81]. Finally, several characteristics of marine natural products, such as halogenation, oxygenation substitution patterns, different degrees of carbon framework functionalization, and stereochemical diversity make chemical synthesis even more complex and challenging for organic chemists. The fulfilment of the principles of green chemistry including the application of atom-economical and high-yield reactions, the use of affordable and nontoxic starting materials and solvents, or the design of protecting group-free chemical synthesis are additional issues to also consider [2,75,82,83]. Thus, novel synthetic strategies have been developed recently, with new scientific and technological progress and insights, and are oriented toward total syntheses of natural products and their stereochemical congeners, at the same time complying with green chemical approach [2].

### 4.1. Synthetic Approaches toward the Synthesis of Terpenoids Isolated from Eunicella Species

Several studies have been conducted over the past two decades with the aim of developing a synthetic approach to obtain highly oxidized eunicellin-type diterpenes [84,85,86,87,88,89]. Two major issues had to be overcome in constructing the tricyclic skeleton of eunicellins. The first was stereoselective formation of hydroisobenzofuran core, which possesses four stereogenic centers and the second was formation of the nine-membered ring. Approaches, including a first synthesis of the isobenzofuran part followed by the cyclization of the nine-membered ring or vice versa, have been developed [88].

**Scheme 1:**
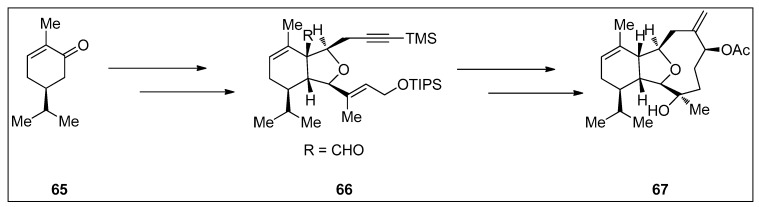
MacMillian’s and Overman’s total synthesis of cladiellin (**67**) [90].

The first successful total synthesis of cladiellin, (−)-7-deacetoxyalcyonine (**67**), starting from (*S*)-dihydrocarvone **65**, was accomplished in 1995 by MacMillian and Overman. Prins-pinacol rearrangement was employed to achieve hydroisobenzofuran **66** as a single diastereomer, followed by Nozaki–Hiyama–Kishi coupling reaction, resulting in the final tricyclic product (Scheme 1) [90]. Since that, numerous total or partial syntheses of eunicellins (cladiellins) and other types of C2–C11 cyclized cembranoids were conducted, which had been previously reviewed by Ellis et al. in 2008 [89].

**Scheme 2:**
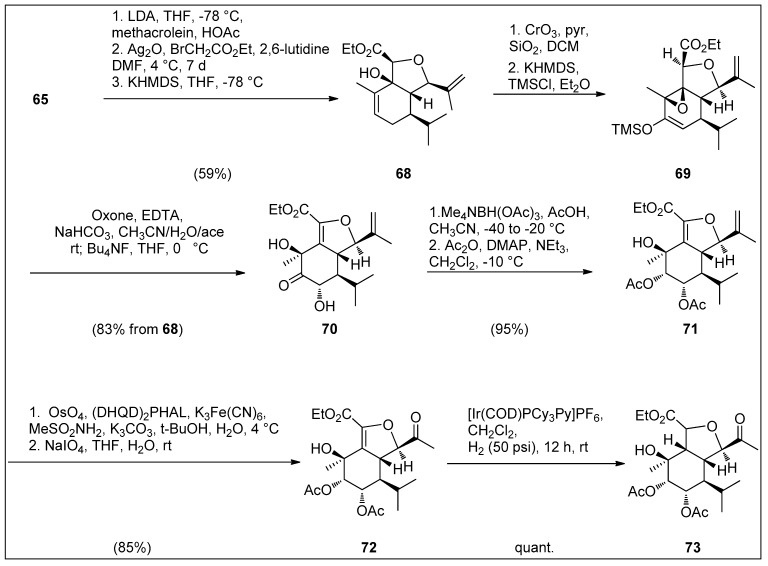
McIntosh’s synthetic approach toward the hydroisobenzofuran core (**73**) of the massileunicellins [85,86].

Synthetic access to eunicellin-type metabolites produced by members of the genus *Eunicella* needs still to be developed. Up to now, profound investigations were performed by the McIntosh group to evolve the complete hydroisobenzofuran core from less oxidized compounds, thus obtaining the more complex stereochemistry of massileunicellins [86,91]. They synthesized hydroisobenzofuran of massileunicellins containing eight of a total of nine stereocenters in an overall 12 steps with a single temporary trimethylsilylation protection of hydroxyl group. As shown in Scheme 2, (*S*)-(+)-carvone (**65**) was reacted with methacrolein in aldol reaction, followed by Williamson etherification and cycloaldolization of the formed glycolate intermediate to afford allylic alcohol **68** in 85% yield [87,91]. The intermediate **68** is converted to epoxy ketone with Collins’ reagent, which in subsequent treatment with potassium bis(trimethylsilyl)amide/trimethylsilyl chloride (KHMDS/TMSCl) yielded silylated enolate **69**. Rubottom oxidation by using dioxirane produced keto diol **70** in 83% yield from intermediate **68**. Evans–Saksena reduction of product **70** by using borohydride reagent led to the corresponding triol, which was subsequently esterified to diacetate **71** in high yield as a single diastereomer. To obtain the desired hydroisobenzofuran product **73**, **71** was subjected to dihydroxylation followed by oxidative cleavage with NaIO_4_, giving rise to methyl ketone **72**. Finally, hydrogenation of a double bond of **72** was conducted with Crabtree’s catalyst at moderate H_2_ pressure to yield quantitively the hydroisobenzofuran core **73** [87].

**Scheme 3:**
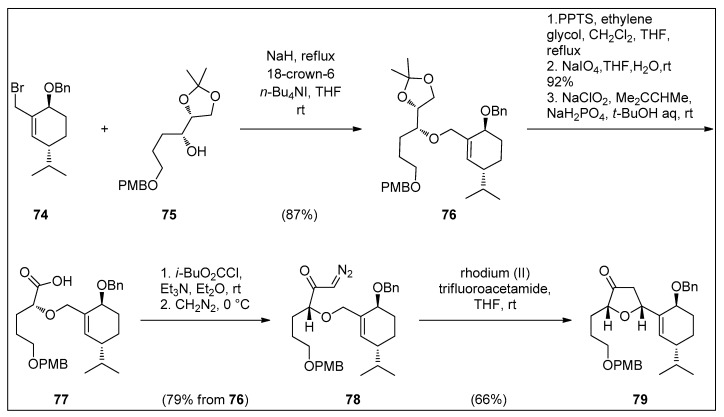
Clark’s synthesis of dihydrofuranone (**79**) via intramolecular dihydrofuranone formation through diazo ketone (**78**) [85].

Clark’s group previously developed synthetic approaches to synthesize the tricyclic core of labiatin A, which is an unusual eunicellin (cladiellin) possessing a C2–C6 ether bridge as discussed above (vide infra) [84,85,92,93]. The authors firstly synthesized bromide **74** in five steps and coupled it through Williams’s etherification with alcohol **75** that was easily prepared from d-mannitol (Scheme 3). The obtained ether **76** (87%) was deprotected with pyridinium *p*-toluensulfonate (PPTS) to cleave the acetonide moiety, and the produced syn-diol was oxidatively cleaved with NaIO_4_ to form a terminal aldehyde which was further oxidized to carboxylic acid **77** with NaClO_2_. A mixed anhydride formation of acid **77** and chloroformate followed by subsequent treatment with diazomethane gave the diazo ketone **78** in 79% yield from **76**. Finally, cyclization of diazo compound **78** was performed by using rhodium (II) tifluoroacetamide as a catalyst and produced dihydrofuranone **79** in 66% yield [85].

**Scheme 4:**
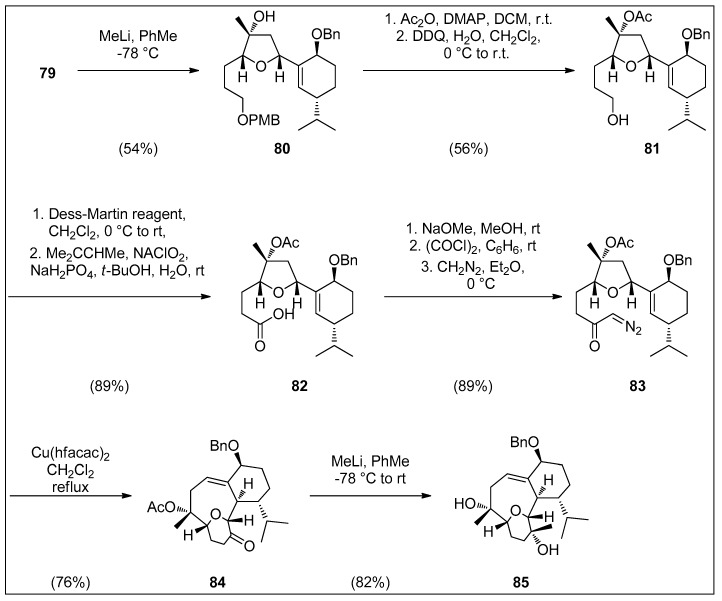
Synthesis of the tricyclic core (**85**) of labiatin A (**11**).

Dihydrofuranone **79** was reacted with methyl lithium giving rise to tert-alcohol **80** (85%) (Scheme 4). Acetylation and removal of *p*-methoxybenzyl protecting group (PMB) with 2,3-dichloro-5,6-dicyano-1,4-benzoquinone (DDQ) of **80** produced primary alcohol **81** (56%). The latter was converted to carboxylic acid **82** (89%) after Dess–Martin and Pinick type oxidation reactions. The **82** was transformed to the corresponding acyl chloride with oxalyl chloride, and, after sequential treatment with diazomethane, resulted in the formation of diazo ketone **83** (89%) required for the final key step. Intramolecular cyclization of the electrophilic carbenoid species of intermediate **83** with copper (II) hexafluoroacetylacetonate gave tricyclic ketone **84** (76%). Finally, after 21 steps in total, tertiary alcohol **85** was formed by the reaction with MeLi leading to the carbon core of labiatin A (**11**) as a single isomer. Its structure was confirmed by X-ray crystallography [85].

### 4.2. Synthesis of Granulatamide Alkaloids

**Scheme 5:**
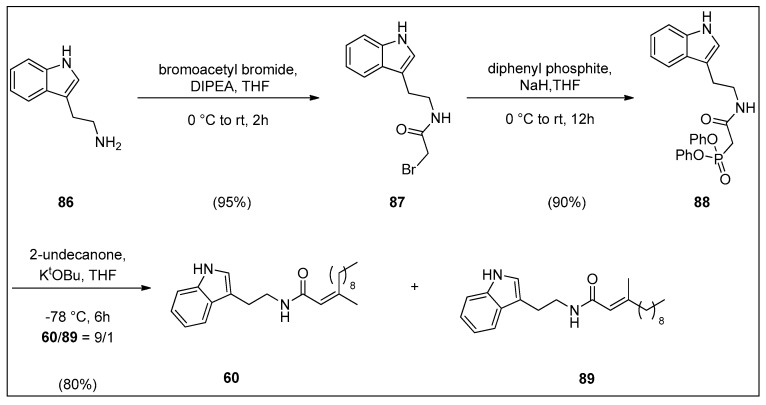
Pakhare’s and Kusurkar’s synthesis of granulatamide A (**60**) via *Z*-favored Horner–Wadsworth–Emmons olefination [94].

In order to obtain the kinetically favored *Z*-isomer of cytotoxic alkaloids, Pakhare and Kusurkar reported the first total synthesis of granulatamide A (**60**) from tryptamine (**86**) via Horner–Wadsworth–Emmons olefination reaction (Scheme 5). In brief, tryptamine (**86**) was converted to bromoacetamide **87** which was treated with diphenyl phosphite under basic conditions to provide phosphonate **88** in 90% yield. The desired natural product granulatamide A (**60**) and its *E*-congener **89** were obtained by the reaction of phosphonate **88** with 2-undecanone in E/Z ratio = 9/1 and 80% overall yield. [94].

**Scheme 6:**
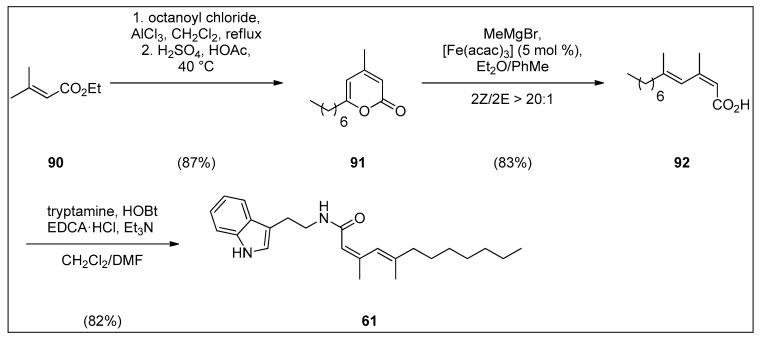
Sun’s and Fürstner’s Fe-catalyzed synthesis of granulatamide B (**61**) via formal ring-opening/cross-coupling reaction of 2-pyrones [95].

Sun and Fürstner developed a highly yielded three-step total synthesis of the cytotoxic tryptamine derivative granulatamide B (**61**) by employing their ring-opening/cross-coupling reaction of 2-pyrones methodology (Scheme 6). Pyrone **91** was obtained in multigram amounts by the reaction of the commercial crotonate **90** and octanoyl chloride. The iron catalyzed ring-opening/cross-coupling of pyrone **91** with methyl Grignard reagent smoothly afforded the acid92 as a single isomer in 83% yield. The amide bond formation was performed under standard conditions by using 1-hydroxy benzotriazole/1-ethyl-3-[3-dimethylaminopropyl] carbodiimide hydrochloride (HOBt/EDCA) coupling protocol to furnish granulatamide B (**61**) [95].

## 5. Conclusions

Natural products and their semisynthetic derivatives have been traditionally among the most important sources in the creation of novel products, especially in the pharmaceutical and cosmetic industries.

As shown in this review, the interdisciplinary studies linked to gorgonian soft corals have contributed to the discovery of new marine natural products. More than 60 metabolites were isolated from the *Eunicella* species in the past three decades, belonging to the class of terpenoids, sterols, alkaloids and nucleosides. These metabolites exhibited anticancer, anti-inflammatory, antiviral, analgesic, and other activities. Their full therapeutic spectra remain to be examined in detail. Already, seven compounds displayed very high cytotoxic activities against cancer cell lines in vitro, namely palmonine B (**3**), labiatin C (**12**), labiatin D (**13**), secosterols **38** and **39**, and granualtamides A (**60**) and B (**61**) with IC_50_ values less than 10 μM or 10 μg/mL. Epidioxysterol **48** showed significant immunosuppressive activity, while nucleosides **62** and **64** showed relevant antiviral activity against DNA viruses.

The interesting biological activities of *Eunicella’*s secondary metabolites have attracted a broad attention of the scientific community with the purpose to isolate novel compounds, develop new and innovative synthetic methodologies, or conduct their partial or total synthesis. By committing studies in the direction of their synthesis, essential stereochemical and mechanistic issues were encountered and solved. The partial or total synthesis of these natural products is expected to guide advancements of new chemical approaches with wider synthetic applications. In conclusion, molecules isolated from the genus *Eunicella*, from their first discovery to date, present an interesting case which has stimulated research advancements in diverse scientific fields, attaining new valuable experimental and theoretical knowledge for future development in medicinal applications.

## Figures and Tables

**Table 1:** The most promising diterpenoids with remarkable cytotoxic activity isolated and studied from corals of the genus *Eunicella*.

Compound	Name	Source	Tumor Cell Line	IC_50_/(µg/mL)	Reference
**3**	Palmonine B	*E. verrucosa*	P-388 MEL28	5 5	[55]
**5**	Palmonine D	*E. verrucosa* *E. singularis*	MCF-7	49	[19]
**7**	Palmonine F	*E. verrucosa* *E. singularis*	MCF-7	13	[19]
**12**	Labiatin B	*E. labiata*	HCT-116	0.85	[26]
**13**	Labiatin C	*E. labiata*	NSCLC-N6	7.7	[22]
**15**	Labiatin E	*E. labiata*	NSCLC-N6	35	[22]

**Table 2:** The most promising sterols with remarkable cytotoxic activity isolated and studied from the genus *Eunicella*.

Compound	Class	Source	Tumor Cell Line	IC_50_ *	Reference
**38**	Secosterol	*E. cavolini*	MCF-7 HeLa	7.6 ^a^ 28.1 ^a^	[34] [38]
**39**	Secosterol	*E. cavolini*	MCF-7	7.4 ^a^	[34]
**43**	Secosterol	*E. cavolini* *S. suberosa*	HeLa	23.3 ^a^	[38]
**45**	Epidioxysterol	*E. cavolini* *Topsentia* sp.	A549 SK-OV-3 SK-MEL-2 HCT15	30.0 ^b^ 19.4 ^b^ 25.9 ^b^ 21.4 ^b^	[62]
**47**	Epidioxysterol	*E. cavolini* *Monanchora* sp.	A-498	23 ^a^	[63]
**48**	Epidioxysterol	*E. cavolini* *E. singularis* *M. azedarach* *H. sphaerocarpa*	A549 H460 HGC27 A2780	10.9 ^b^ 10.9 ^b^ 11.6 ^b^ 16 ^b^	[64,65]
**59**	Sterol	*E. singularis*	MCF-7	30 ^b^	[19]

* Values for IC_50_ are expressed as either μM (a) or μg/mL (b).

**Table 3:** Growth inhibitory activity of granulatamides A (**60**) and B (**61**) against various tumor cell lines [51].

Compound	IC_50_/µM						
	Du-145	LN-caP	SK-BR3	HT29	IGROV	A549	K-562
**60**	1.7	4.7	2.7	2.2	6.7	6.7	6.8
**61**	7.7	3.5	6.0	>10	8.2	8.9	4.3

## List of Abbreviations

A2780	human ovarian carcinoma cell line
A498	human renal epithelial cancer cell line
A549	human lung carcinoma cell line
ASL	acetylsalicylate of lysine
COX-2	cyclooxygenase-2
DDQ	2,3-dichloro-5,6-dicyano-1,4-benzoquinone
(DHQD)2PHAL	hydroquinidine 1,4-phthalazinediyl diether
DIPEA	N,N-diisopropylethylamine
DMAP	4-dimethylaminopyridine
DMF	dimethylformamide
Du145	human prostate cancer cell line
EDTA	ethylenediaminetetraacetic acid
H460	human large cell lung cancer cell line
HCT-115	human colorectal adenocarcinoma cell line
HCT-116	human colon cancer cell line
HeLa	human cervical cancer cell line
HepG2	human hepatocellular carcinoma cell line
Hfacac	hexafluoroacetylacetonat
HGC-27	human gastric cancer cell line
HK-2	human papillomavirus 16 (HPV-16) transformed renal cell line
HL-60	human leukemia cell line
HT29	human colon adenocarcinoma cell line
HTLV-1	human T-lymphotropic virus 1
IC50	half maximal inhibitory concentration
IGROV	human ovarian cancer cell line
iNOS	inducible nitric oxide synthase
K562	human myelogenous leukemia cell line
KB	keratin-forming tumor cell line HeLa
KHMDS	potassium bis(trimethylsilyl)amide
KtOBu	potassium t-butoxide
L1210	murine lymphocytic leukemia cell line
LDA	lithium diisopropylamide
LNcaP	androgen-sensitive human prostate adenocarcinoma cell line
LPS	lipopolysaccharide
MCF-7	human breast adenocarcinoma cell line
MES-SA	human uterine carcinoma cell line
MIC50	minimum inhibitory concentration required to inhibit the growth of 50% or organisms
NO	nitric oxide
NSCLC-N6	human non-small cell lung cancer cell line
P-388	menogaril-resistant leukemia cell line
PPTS	pyridinium p-toluensulfonate
SGC-7901	human gastric cancer cell line
SK-BR3	human breast cancer cell line
SK-MEL-2	human melanoma cell line derived from metastasis on skin of thigh
SK-OV-3	human ovarian carcinoma cell line
THF	tetrahydrofuran
TMSCl	trimethylsilyl chloride
Vero	epithelial cell line derived from kidney of Cercopithecus aethiops
XF498	human glioblastoma cell line
